# Inventory study of non-tuberculous mycobacteria in the European Union

**DOI:** 10.1186/1471-2334-14-62

**Published:** 2014-02-06

**Authors:** Marieke J van der Werf, Csaba Ködmön, Vera Katalinić-Janković, Tiina Kummik, Hanna Soini, Elvira Richter, Dimitrios Papaventsis, Enrico Tortoli, Monique Perrin, Dick van Soolingen, Manca Žolnir-Dovč, Vibeke Østergaard Thomsen

**Affiliations:** 1 European Centre for Disease Prevention and Control, Stockholm, Sweden; 2 National Mycobacterium Reference Laboratory, Croatian National Institute of Public Health, Zagreb, Croatia; 3 Department of Mycobacteriology, Tartu University Hospital, Tartu, Estonia; 4 National Institute for Health and Welfare, Turku, Finland; 5 National Reference Laboratory for Mycobacteria, Forschungszentrum Borstel, Borstel, Germany; 6 National Reference Laboratory for Mycobacteria, “Sotiria” Chest Diseases Hospital, Athens, Greece; 7 Emerging Bacterial Pathogens Unit, San Raffaele Scientific Institute, Milan, Italy; 8 Division de Bactériologie – Parasitologie, Laboratoire National de Santé, Luxembourg, Luxembourg; 9 National Institute for Public Health and the Environment (RIVM), Bilthoven, The Netherlands; 10 National Reference Laboratory for Mycobacteria, University Clinic of Respiratory and Allergic Diseases, Golnik, Slovenia; 11 International Reference Laboratory of Mycobacteriology, Statens Serum Institut, Copenhagen, Denmark

**Keywords:** Non-tuberculous mycobacteria, Mycobacterioses, Epidemiology, European Union

## Abstract

**Background:**

Since non-tuberculous mycobacteria (NTM) disease is not notifiable in most European Union (EU) and European Economic Area (EEA) countries, the epidemiological situation of the >150 NTM species is largely unknown. We aimed to collect data on the frequency of NTM detection and NTM species types in EU/EEA countries.

**Methods:**

Officially nominated national tuberculosis reference laboratories of all EU/EEA countries were asked to provide information on: laboratory routines for detection and identification of NTM, including drug sensitivity testing (DST) methods; data on the number and type of NTM species identified; coverage and completeness of the provided data on NTM; type and number of human specimens tested for NTM; and number of specimens tested for Mycobacterium tuberculosis complex and NTM. This information was summarized and the main results are described.

**Results:**

In total, 99 different NTM species were identified with *M. avium, M. gordonae, M. xenopi , M. intracellulare, and M. fortuitum* identified most frequently. Seven percent of the NTM species could not be identified. NTM was cultured from between 0.4-2.0% of the specimens (data from four countries). The laboratories use culturing methods optimised for *M. tuberculosis* complex. Identification is mainly carried out by a commercial line probe assay supplemented with sequencing. Most laboratories carried out DST for rapid growers and only at the explicit clinical request for slow growers.

**Conclusion:**

It is likely that the prevalence of NTM is underestimated because diagnostic procedures are not optimized specifically for NTM and isolates may not be referred to the national reference laboratory for identification. Due to the diagnostic challenges and the need to establish the clinical relevance of NTM, we recommend that countries should concentrate detection and identification in only few laboratories.

## Background

Today, more than 150 non-tuberculous mycobacteria (NTM) species have been described [1-3]. Many have only been described after the introduction of molecular methods in the 1990’ies and still quite a number of NTM remain unclassified. NTMs are capable of causing disease, but the pathogenicity varies among the species [4]. In general, it is a challenge to identify NTM species as the specific causal agent of disease, especially because recovery of NTM from a culture of a respiratory specimen may be due to contamination of the specimen or transient colonization of the patient. Therefore the clinical significance of detected NTM in non-sterile specimens needs to be evaluated for the individual patient, which is usually done according to the guidelines from the American Thoracic Society (ATS) [5].

So far, there is very limited evidence for person-to-person transmission of NTM [1,6,7]. Outbreaks caused by exposure to the same reservoir have been described [8]. It is generally accepted that a wide range of animal and environmental sources (swimming pools, aquaria) are reservoirs for NTM. Examples include marine animals for *Mycobacterium marinum*, tap waters and potting for *M. avium* complex, and tap water for *M. gordonae*. NTM species have also been detected in medical devices in hospital settings [9] and can therefore pose a risk to vulnerable hospitalized patients. For most species, no single reservoir has been identified.

Although it is a challenge to link NTM to disease in man, significant NTM disease has been described among patients suffering from cystic fibrosis (often with *M. abscessus*) [7], chronic obstructive pulmonary disease (COPD) [10], HIV and other immunosuppressive conditions [11], and in otherwise healthy children having cervical NTM lymph adenitis [12]. In addition, inhaled corticosteroids [13] and TNF-a inhibitor treatment [14,15] are associated with NTM disease. Host factors such as IL-12 deficiency has been described among other genetic host factors [16]. NTM infections are most frequently located in the lungs, but may also be found in lymph nodes, skin, soft tissue, and joint and bones, and can be disseminated [17].

NTM disease is not notifiable in most countries. Therefore, the overall epidemiological situation of NTM in the European Union (EU) and European Economic Area (EEA) is largely unknown. Several EU countries have published reports about the situation in their country [18]. In England, Wales and Northern Ireland an increase in NTM was reported [19]. A report from Portugal found a high percentage of NTM strains among mycobacterial isolates [20]. A multi country overview that included information from EU/EEA countries with data up to 1996 was published by the Working Group of the Bacteriology and Immunology Section of the International Union Against Tuberculosis and Lung Disease [21]. This study concluded that there was an increase in the number of NTM isolated from clinical samples of patients. A recently published snapshot of NTM in pulmonary samples collected in 2008 in 30 different countries from different continents showed that *M. avium* complex (MAC) bacteria predominated in most countries, followed by *M. gordonae* and *M. xenopi *[22].

The aim of the present study was to provide an overview on the frequency of NTM detection and the types of NTM species detected in EU/EEA countries. In addition, an overview of the laboratory methods used for detection and identification of NTM in EU/EEA countries is given.

## Methods

We invited all EU/EEA countries to participate in this inventory by contacting the European Centre for Disease Prevention and Control (ECDC) national TB reference laboratory contact points in August 2012. Countries were asked to provide information on: laboratory routines for detection and identification of NTM, including drug sensitivity testing (DST) methods; data on the number and type of NTM species identified in pulmonary as well as non-pulmonary specimens; coverage and completeness of the provided data on NTM; type and number of human specimens tested for NTM; and number of specimens tested for *Mycobacterium tuberculosis* complex and NTM.

The provided information was summarized in tables and the main results are described.

## Results

Ten EU countries, Croatia, Denmark, Estonia, Finland, Germany, Greece, Italy, Luxembourg, Netherlands, Slovenia, provided data for the inventory study on NTM (Table 1). In 6 countries (Croatia, Denmark, Estonia, Finland, Luxembourg, Slovenia), the NTM data are considered to be complete for the country. In Greece and the Netherlands, the data are considered almost complete, and in Italy the data are complete for a part of the country. The National Reference Laboratory of Germany only receives a fraction of the NTM samples and thus the data are incomplete. Finland is the only participating country in which notification of NTM is mandatory. The data in Table 2 refer to positive specimens for Croatia, Estonia, Germany, Greece, Luxembourg and Slovenia, whereas for Denmark, Finland, Italy and the Netherlands the data refer to patients with NTM.

**Table 1:** Detection, identification and drug sensitivity testing procedures for non-tuberculous mycobacteria (NTM) in 10 European Union countries

**Country**	**Detection NTM**	**Identification NTM**	**Drug sensitivity testing**
Croatia	Smear microscopy and culture on solid and liquid media	GenoType Mycobacterium CM/AS (Hain Lifescience, Nehren, Germany) supplemented with phenotypic analysis including examination of growth characteristics, temperature tolerance, pigmentation and biochemical properties	For rapidly growing species* by E-test (AB Biodisk, Solna, Sweden) strips or minimum inhibitory concentration (MIC) testing.
Denmark	Smear microscopy, culture on solid (Löwenstein-Jensen) and liquid media (BD Bactec MGIT 960) and PCR	GenoType Mycobacterium CM/AS (Hain Lifescience, Nehren, Germany) supplemented by 16S rDNA sequencing and/or other methods	For rapid growing species: MIC determination on TREK microwell plates (TREK Diagnostic Systems, Cleveland, Ohio, United States of America). For slow growing species only at specific request of clinician
Estonia	Smear microscopy, culture on solid media (e.g. Löwenstein-Jensen) and liquid media (BD Bactec MGIT 960)	GenoType Mycobacterium CM/AS (Hain Lifescience, Nehren, Germany)	Not performed
Finland	Culture on solid media (e.g. Löwenstein-Jensen or Middlebrook 7H11) and liquid media (BD Bactec MGIT 960)	GenoType Mycobacterium CM/AS (Hain Lifescience, Nehren, Germany) and, if needed, 16S rDNA sequencing	For species belonging to *M. avium* complex (MAC): narrow range MIC method for azithromycin, clarithromycin, ethambutol, clofatzimin, and rifabutin. For rapid growing species belonging to the *M. chelonae-fortuitum* complex: E-test (BioMerieux, Lyon, France)
Germany	Smear microscopy and culture on solid and liquid media, and for certain specimens (skin, lymph nodes, sputum from CF patients) incubation on specific media at lower temperatures (31°C)	Line probe assays or sequence analysis(16S rDNA, ITS, *rpo*B)	Slowly growing species: modified proportion method using MGIT liquid media (BACTEC MGIT 960 or manual system). Drugs tested comprise first line TB drugs as well as clarithromycin or on special request fluoroquinolones. For other slow growing species, i.e*. M. avium/intracellulare*: A combination of drugs is applied for DST testing
Greece	Smear microscopy and culture on solid and liquid media	GenoType Mycobacterium CM/AS (Hain Lifescience, Nehren, Germany) and when requested by the clinicians, 16S rDNA and hsp65 gene sequencing	For slow growing species, the modified proportion method using BACTEC MGIT 960 or LJ is applied. First line anti-TB drugs and clarithromycin are tested according to ATS/ IDSA guidelines [5]. On special request FQs are also tested. For rapid growing species, MIC testing on microwell plates is performed (TREK Diagnostic Systems, Cleveland, Ohio, United States of America).
Italy	Culture on solid (Löwenstein-Jensen) and liquid media (BD Bactec MGIT 960)	GenoType Mycobacterium CM/AS (Hain Lifescience, Nehren, Germany) and sequencing of genetic regions (16S rRNA and/or *hsp65* genes for slow growers, *rpoB* gene for rapid growers, ITS1 for members of MAC) whenever the commercially available molecular probes (Genotype CM and AS) produced an identification known to be not 100% free from cross reactions with other species	For rapid growing species: MIC determination (Rapidly growing mycobacteria panel, TREK Diagnostic Systems (Cleveland, Ohio, United States of America). For slow growing species: susceptibility to chlarithromycin is determined for some cases.
Luxembourg	Culture on solid (Lowenstein Jensen, Coletsos and Middlebrook 7H11) and liquid media (BD Bactec MGIT 960)	To distinguish MTB complex from NTM isolates the Anyplex MTB/NTM Real Time detection kit (Seegene) is used. In a second stage, NTM specimens are sequenced (sequencing of the hsp65 gene)	Not performed in country. On explicit request of the doctor, the isolate is sent to the Belgian Scientific Institute for Public Health
Netherlands	Culture on liquid media (BD Bactec MGIT 960)	Commercially available reverse line blot assays	7H10 agar dilution method [23] for both slow and rapid growing species
Slovenia	Culture on solid and liquid media. Some clinical samples are incubated at 30°C and on media with special supplements.	GenoType Mycobacterium CM/AS (Hain Lifescience, Nehren, Germany), colony morphology on transparent medium Middlebrook 7H10, and some phenotypic tests	At specific request of clinician for rapid growing species and some slow growing species: E-test (AB Biodisk, Solna, Sweden)

*For definition of slow growing and rapidly growing species see reference [24].

**Table 2:** Species distribution of non-tuberculous mycobacteria isolated in 10 European Union countries

Species*	**Country**										**Total**
	**Croatia (2008-2010)**	**Denmark (2011)**	**Estonia (2004-2011)**	**Finland (1995-2011)**	**Germany (2011)**	**Greece (2005-2011)**	**Italy (2001-2010)**	**Luxembourg (2009-2011)**	**Netherlands (2006-2011)**	**Slovenia (2000-2010)**	
*M. avium*	33	86	308	1,568	176	101	350	23	1,274	349	4,268
*M. gordonae*	377	9	96	1,286	176	31	253	26	623	365	3,242
*M. xenopi*	122	7	12		13	9	687	13	77	615	1,555
*M. intracellulare*	23	15	52	636	272	49	104	3	359	156	1,669
*M. fortuitum*	98	2	65	521	68	66	144		239	150	1,353
*M. kansasii*	8	5	11	53	58	22	39	6	283	181	666
*M. chelonae*	31	3	20	179	56	37	55	4	173	32	590
*M. abscessus*	25	10	17	94	37	17	111	11	228	24	574
*M. malmoense*		12	14	184	10	7	11	6	108	22	374
*M. lentiflavum*	6		5	246	4	6	45		5		317
*M. marinum*		7	3	50	29	5	15	4	115	6	234
*M. terrae*	52			151			16		6		225
*M. peregrinum*	9	3	15	81		20	33	1	6		168
*M. simiae*		3		69	5		21	3	64		165
*M. bohemicum*				106	1		4	3	10		124
*M. chimaera*							48	5	68		121
*M. interjectum*	1		3	87	6		9		14		120
*M. nonchromogenicum*	18			38	1		41		1	21	120
*M. arupense*					7		83	3	8		101
*M. celatum*	3	7	1		3	8	12	1	11	21	67
*M. mucogenicum*	3				6		38		15		62
*M. scrofulaceum*	2		16				12		12	12	54
*M. szulgai*		2	3		13		11		17	6	52
*M. kumamotonense*					1		46		1		48
*M. heraklionense*							46				46
*M. genavense*					8		5		18		31
*M. phlei*	4				7		3		13		27
*M. vulneris*					26		1				27
*M. haemophilum*					1		1		18		20
*M. triviale*	15						2				17
*M. arosiense*							15				15
*M. florentinum*					1		6	5			12
*M. nebraskense*					11		1				12
*M. neoaurum*					3		5		4		12
*M. vaccae*	10						1				11
*M. elephantis*							10				10
*M. insubricum*					1		8				9
*M. parascrofulaceum*					4		5				9
*M. sherrisii*							9				9
*M. alvei*							2		6		8
*M. porcinum*							5	2	1		8
*M. europaeum*					3		4				7
*M. flavescens*	6						1				7
*M. shimoidei*							4		3		7
*M. gilvum*							6				6
*M. longobardum*							6				6
*M. paraffinicum*					1		4		1		6
*M. triplex*							6				6
*M. branderi*		1			1				3		5
*M. goodii*					1		1		3		5
*M. holsaticum*					2		2		1		5
*M. smegmatis*							4		1		5
*M. thermoresistibile*	1						4				5
*M. cosmeticum*							1		3		4
*M. heckeshornense*		1			1		1		1		4
*M. agri*		1							2		3
*M. brumae*							2		1		3
*M. flavescens/ M. novocastrense*					3						3
*M. frederiksbergense*							3				3
*M. hassiacum*					1				2		3
*M. heidelbergense*					1				2		3
*M. lepraemurium*					3						3
*M. mantenii*							2		1		3
*M. noviomagense*							1		2		3
*M. palustre*							3				3
*M. setense*							3				3
*M. wolinskyi*					1				2		3
*M. aichiense*							2				2
*M. engbaekii*							2				2
*M. gastri*							2				2
*M. immunogenum*							2				2
*M. intermedium*							2				2
*M. llatzerense*							2				2
*M. mageritense*									2		2
*M. monacense*							2				2
*M. novocastrense*							1		1		2
*M. phocaicum*					1		1				2
*M. pulveris*							2				2
*M. rhodesiae*							2				2
*M. riyadhense*					1		1				2
*M. asiaticum*									1		1
*M. aubagnense*					1						1
*M. chlorophenolicum*	1										1
*M. colombiense*							1				1
*M. conceptionense*										1	1
*M. conspicuum*									1		1
*M. duvalii*							1				1
*M. fluoroanthenivorans*					1						1
*M. gadium*							1				1
*M. hiberniae*							1				1
*M. kyorinense*					1						1
*M. massiliense*								1			1
*M. parmense*							1				1
*M. poriferae*							1				1
*M. senegalense*							1				1
*M. senuense*							1				1
*M. timonense*								1			1
*M. tokaiense*							1				1
*M. tusciae*							1				1
Unknown		8	57	429#	41	48	96	2	186	279	717
Total	848	182	698	5,778	1,068	426	2,498	123	3,996	2,240	17,857

*Croatia, Estonia, Germany, Greece, Luxembourg and Slovenia report positive specimens, Denmark, Finland, Italy and the Netherlands report patients with NTM.

#Unknown species and rare species.

In most countries NTM are detected by smear microscopy and by both solid and liquid culture (Table 1). In Denmark, in Finland in one clinical laboratory, and in Luxembourg PCR is used for detection. In Germany PCR is used on special request and in the Netherlands PCR is used by specific laboratories. Denmark, Croatia, Estonia, Finland, Germany, Italy, Luxembourg, the Netherlands, and Slovenia reported to incubate some types of specimens (for example skin and lymph node samples, and sputum from cystic fibrosis patients) at a lower temperature, i.e. 30-32°C. The GenoType Mycobacterium CM/AS (Hain Lifescience, Nehren, Germany) was the most frequent used method for identification of NTM species. Many countries also used sequencing to identify species. Different methods were used for determining the drug sensitivity of the NTM species and often DST was only performed at specific request of a clinician.

*M. avium, M. gordonae, M. xenopi* , *M. intracellulare,* and *M. fortuitum* were detected most frequently overall (Table 2). These species account for two thirds of all identified species. Almost 7% of the isolates could not be identified as an officially recognised species. The rest of the isolates were distributed over 94 species - of which many were observed very infrequently.

The order of the most frequently reported species changes slightly if only data from the four countries that report NTM in patients were taken into account, i.e. *M. avium*, *M. gordonae*, *M. intracellulare*, *M. fortuitum,* and *M. xenopi*.

In Table 2 we show all data provided by the participating countries. Some countries provided data for 1 year only, while others provide data for up to 17 years (Finland). To give each country equal weight we calculated the average number of species per year for countries that provided data for more than one year (data not shown). This weighing procedure did not change the top five most frequent species. It did change the order slightly into *M. avium*, *M. gordonae, M. fortuitum, M. xenopi,* and *M. intracellulare*.

NTM were detected from a variety of clinical specimens as demonstrated by data from Croatia, Italy, Luxembourg and Slovenia in Table 3. Both sterile and non-sterile specimens were reported NTM culture positive. A high number of pulmonary specimens are found NTM culture positive, but this likely reflects the fact that these specimens are submitted for culture more frequently compared to other specimens.

**Table 3:** Specimen types from which non-tuberculous mycobacteria were isolated*

**Specimen type**	**Number of specimen positive for NTM**
Sputum	2,839
Other pulmonary	1,160
Bronchial aspirate	191
Urine	117
Biopsy	74
Blood	47
Stool	45
Other body fluid	28
Gastric lavage	22
Lymph node aspiration	20
Pleural fluid	14
Cutis	8
Cerebrospinal fluid	3
Not specified	2,276

*Data from Croatia, Italy, Luxembourg and Slovenia.

Five countries provided information on the number of specimens tested and the frequency of *M. tuberculosis* complex and NTM isolation (Table 4). Between 2.8 and 11.3% of all tested specimens were positive for *M. tuberculosis* complex. Whereas only between 0.4 and 2.0% tested positive for NTM.

**Table 4:** **Number of specimens tested and specimens positive for *M. tuberculosis *** **complex and non-tuberculous mycobacteria (NTM) in Estonia, Greece, Croatia, Luxembourg, and Slovenia**

**Country**	**Specimens tested, N**	**Specimens positive for *M. tuberculosis *** **complex, N (%)**	**Specimen positive for NTM, N (%)**
Croatia	166,561	8,375 (5.0)	848 (0.5)
Estonia	83,655	9,475 (11.3)	698 (0.8)
Greece	114,106	3,194 (2.8)	426 (0.4)
Luxembourg	6,113	277 (4.5)	123 (2.0)
Slovenia	148,744	11,368 (7.6)	2,240 (1.5)

## Discussion

NTM disease and detection is not notifiable in most EU/EEA countries and therefore high quality information is not readily available. In the countries that participated in the inventory, the national TB reference laboratories often have a good overview of the frequency of detection of NTM and the distribution of mycobacterial isolates over the different species. The reported data show that *M. avium, M. gordonae, M. xenopi, M. intracellulare,* and *M. fortuitum* are isolated most frequently and that approximately 7% of all isolates cannot be identified as valid species.

In most countries included in this inventory, only few laboratories are performing isolation and identification of NTM. This allows for high quality and standardization of species identification. However, together with the fact that clinical and laboratory diagnosis of NTM is challenging it might result in an underestimation of the true prevalence of NTM because samples/isolates need to be send from the laboratory that performed the initial diagnostic test to the laboratory that is able to do the species identification.

The aim of this inventory study was to collect and present available data from national TB reference laboratories on NTM. We did not prescribe by which methods NTM needed to be isolated and identified and also the year of isolation and population were not specified. It would be interesting to use uniform diagnostic methods, sample population and period of sample collection to be able to compare the prevalence and frequency of NTM in different countries.

Since participation in the inventory was voluntary only part of the EU countries participated, probably countries that have information on NTM available. The 10 countries that participated are located in different parts of the EU, both the North and the South, and also the West and the East. Assuming that the distribution of NTM will not be affected by national borders but by geographical location, the frequency of detection of NTM might well be representative for the EU.

Based on Croatian, Estonian, Greek, Luxembourgish and Slovenian data, NTM was cultured from between 0.4-2.0% of the specimens. As it can be very difficult to discriminate NTM disease from TB based on symptoms and clinical findings, specimens are usually submitted for culture of mycobacteria in general. The laboratories use methods optimised for detection of *M. tuberculosis* complex also for culturing of NTM in general with a few exceptions. Both the difficulty in discriminating NTM disease from TB clinically and the fact that it is impossible to optimise specimen pre-treatment and incubation for more than >150 different NTM species makes this is a reasonable routine that may - however - underestimate NTM. Some patients, such as patients suffering from cystic fibrosis, can be expected to be infected with a certain species (*M. abscessus)* and pre-treatment of specimens optimizing isolation of this species can therefore be a good strategy to improve detection [25]*.* Specific elements of an optimal testing algorithm for *M. tuberculosis* complex as well as all non-tuberculous mycobacteria need to be identified and comparison with current routines needs to be carried out.

Identification of NTM is mainly carried out by a commercial line probe assay supplemented with sequencing. The use of the same commercial method in many countries may help standardisation of NTM identification and supports comparison of results between countries, but it also creates some vulnerability in case of quality problems of the commercial kit [26]. For NTM identification, however, this is a minor problem as long as *M. tuberculosis* can be excluded rapidly and sequencing can be applied.

Seven percent of all NTM isolates could not be recognised as valid species. This group actually consists of two distinct groups, depending on the laboratory were the identification was performed. In laboratories using commercial DNA probes this group consists of all strains that cannot be identified by commercial DNA probes. In other laboratories this group consists of species that had not yet been recognized as validly described species at the moment of identification. Some species may not be so rare if the commercial identification methods would allow for identification. Thus, new methods for identification are needed. Using additional methods for identification, the Netherlands recently described the distinction between *M. noviomagense* and *M. xenopi*, to which it is closely related [27]. *M. xenopi* is clinically significant in a high percentage of the patients whereas *M. noviomagense* has never been associated with NTM disease. Therefore, such a more fine-tuned distinction serves both the clinician and the patient. Whole Genome Sequencing (WGS) will probably facilitate an improved taxonomy of NTM in the future.

Even though NTMs do not seem to be very transmissible from person to person, especially in immune-competent persons, and outbreaks are infrequent, information on the frequency of the disease is useful, especially because the EU/EEA population is aging and more persons may suffer from chronic disease and possible immunosuppression and therefore be at risk for NTM disease. Risk groups for pulmonary NTM disease include patients with COPD, bronchiectasis, previous TB, and those using corticosteroids [13] or undergoing organ transplant [8]. A specific risk groups for pulmonary NTM disease with *M. abscessus* are cystic fibrosis patients [28].

Often the clinical significance of isolation of NTM from non-sterile specimens such as sputum is uncertain, and the clinical, radiography and microbiology criteria of the American Thoracic Society (ATS) are applied to come to a conclusion about the necessity of treatment [5]. A retrospective review of patient files in the Netherlands led to the conclusion that the clinical relevance differed significantly by NTM species [29]. For instance, in most cases where *M. szulgai* was isolated, treatment was needed. In contrast, isolation of *M. gordonae* was hardly ever clinically relevant. Tortoli and Koh also emphasized that NTM species differ in their ability to cause lung disease in humans [30,31] and found that *M. kansasii*, *M. malmoense*, *M. szulgai* and *M. shimoidei*, which are considered more pathogenic than other NTMs, accounted, as a whole, for 1.1% of the isolates. The number of cases in which *M. xenopi* was responsible for severe pulmonary disease, although difficult to quantify, was however considerable, which is in agreement with findings from Denmark that 51% of patients with *M. xenopi* detected are dead after 5 years [32]. In our study, we collected information about frequency of NTM isolation from laboratories in the participating countries. The collected information does not inform whether the patients from which the specimens were collected fulfilled the clinical, radiography and microbiology criteria of the ATS.

Drug susceptibility testing has been standardized for rapid growers and few slowly growing species [33]. A majority of laboratories in this study, carried out DST for rapid growers by either E-test or TREK microbroth dilution. For slowly growing species, a variety of methods were applied. Often DST was carried out only after specific request of the clinician. For many species, questions regarding the correlation between DST results and treatment response remain to be addressed.

## Conclusion

There is likely an under reporting of NTM because it is not notifiable, and in most countries samples/isolates need to be send to the reference laboratory for detection and/or identification of NTM. Also, diagnostic procedures are not optimized for isolation of the different NTM species. Since risk groups that are more prone to NTM disease are growing, it is recommended to systematically collect laboratory information and some basic epidemiological data to monitor NTM at country level.

Due to the large number of different NTM species and the challenge in identification and in establishing the clinical relevance countries should concentrate the diagnosis and species identification in one or few laboratories. Laboratory experts from should be available for consultation by the clinicians about the interpretation of the laboratory result.

## Abbreviations

ATS: American thoracic society; COPD: Chronic obstructive pulmonary disease; DST: Drug sensitivity testing; ECDC: European centre for disease prevention and control; EEA: European economic area; EU: European Union; MAC: *Mycobacterium avium* complex; MIC: Minimum inhibitory concentration; NTM: Non tuberculous mycobacteria.

## Competing interests

The authors declare that they have no competing interests.

## Authors’ contributions

MW conceived of the study, participated in the design and coordination, and drafted and revised the manuscript. CK participated in the design and coordination and helped to draft the tables. VKJ, TK, HS, ER, DP, ET, MP, DS, and MZD provided data, reviewed the draft manuscript, and have given final approval of the version to be published. VØT conceived of the study, participated in the design and coordination, provided data and helped to draft the manuscript. All authors read and approved the final manuscript.

## Pre-publication history

The pre-publication history for this paper can be accessed here:

http://www.biomedcentral.com/1471-2334/14/62/prepub
